# The subunits of the S-phase checkpoint complex Mrc1/Tof1/Csm3: dynamics and interdependence

**DOI:** 10.1186/1747-1028-9-4

**Published:** 2014-10-31

**Authors:** Sonya Dimitrova Uzunova, Alexander Stefanov Zarkov, Anna Marianova Ivanova, Stoyno Stefanov Stoynov, Marina Nedelcheva Nedelcheva-Veleva

**Affiliations:** 1 Institute of Molecular Biology “Roumen Tsanev”, Bulgarian Academy of Sciences, 21 “Acad. George Bonchev” Str., 1113 Sofia, Bulgaria

**Keywords:** Cell cycle, Protein localization, Adaptation, In situ chromatin binding assay, Mrc1, Tof1, Csm3

## Abstract

**Background:**

The S-phase checkpoint aims to prevent cells from generation of extensive single-stranded DNA that predisposes to genome instability. The *S. cerevisiae* complex Tof1/Csm3/Mrc1 acts to restrain the replicative MCM helicase when DNA synthesis is prohibited. Keeping the replication machinery intact allows restart of the replication fork when the block is relieved. Although the subunits of the Tof1/Csm3/Mrc1 complex are well studied, the impact of every single subunit on the triple complex formation and function needs to be established.

**Findings:**

This work studies the cellular localization and the chromatin binding of GFP-tagged subunits when the complex is intact and when a subunit is missing. We demonstrate that the complex is formed in cell nucleus, not the cytoplasm, as Tof1, Csm3 and Mrc1 enter the nucleus independently from one another. Via *in situ* chromatin binding assay we show that a Tof1-Csm3 dimer formation and chromatin binding is required to ensure the attachment of Mrc1 to chromatin. Our study indicates that the translocation into the nucleus is not the process to regulate the timing of chromatin association of Mrc1. We also studied the nuclear behavior of Mrc1 subunit in the process of adaptation to the presence hydroxyurea. Our results indicate that after prolonged HU incubation, cells bypass the S-phase checkpoint and proceed throughout the cell cycle. This process is accompanied by Mrc1 chromatin detachment and Rad53 dephosphorylation.

**Conclusions:**

In *S. cerevisiae* the subunits of the S-phase checkpoint complex Mrc1/Tof1/Csm3 independently enter the cell nucleus, where a Tof1-Csm3 dimer is formed to ensure the chromatin binding of Mrc1 and favor DNA replication and S-phase checkpoint fork arrest. In the process of adaptation to the presence of hydroxyurea Mrc1 is detached from chromatin and Rad53 checkpoint activity is diminished in order to allow S-phase checkpoint escape and completion of the cell cycle.

## Background

The activation of the S-phase checkpoint aims to preserve genome stability when an impediment for strict DNA synthesis arises. It turns on a cascade of events and results in replicational block that provides time for the repair systems to eliminate the problem. Then the ordinary dynamics of the cell cycle is restored and DNA synthesis and segregation completed. A complex of three proteins, named in *S. cerevisiae* Tof1/Csm3/Mrc1, plays a critical role in that process. Those proteins are conserved among organisms. Tof1’s familiar orthologs are Swi1 in *S. pombe* and Timeless (Tim1) in human. Csm3’s orthologs are Swi3 for fusion yeast and Tipin for *H. sapiens* and Mrc1’s are respectively Mrc1 in *S. pombe* and Claspin in higher eukaryotes [1-5]. Today they are categorized as S-phase checkpoint mediators [2]. Mediators act as protein bridges (platforms) that bring together sensor or effector kinases, functioning afterwards in the signal cascades. The three proteins are found to co-localize at normal and stalled replication forks [5,6]. They co-precipitate together in dynamic and stalled replication forks [7], suggesting that the three proteins form a complex. Tof1, Csm1 and Mrc1 also co-precipitate with subunits of the MCM replicative helicase both from exponentially growing culture and arrested by hydroxyurea (HU) one [7-9]. The synthetic lethality between deletions of *tof1*, *csm3* or *mrc1* and mutations in polymerase α/primase complex, shows that the products of these genes are interdependent in order to guarantee the correctness of the replication process [7]. These dependencies suggest that the Tof1/Csm3/Mrc1 complex aims to keep together the polymerase and helicase in order to prevent lethality of cells, when DNA synthesis is compromised.

It was shown that lack of Mrc1 leads to MCM-Cdc45 and Pol ϵ separation [9-11]. A function to stabilize the forks for Mrc1 and its orthologs was suggested [11,12]. Another *S. cerevisiae* protein – Ctf4 (Mcl1 in *S. pombe* and And1 in *X. laevis* and *H. sapiens*) [13-17] was also found to be involved in cell cycle progression and sister chromatide cohesion [18,19]. It physically interacts with GINS and Pol α at replication progressing complex (RPC) [20-25]. Recent data demonstrate that the ortholog of Ctf4 - And1 from *Xenopus* egg extract binds Tipin, and their binding is necessary for the stable Pol α association to chromatin under unchallenged conditions [26]. Indirect binding to Mrc1 was also revealed [27] and was demonstrated that this binding is sufficient for E3 ubiquitine ligase SCF^Dia2^ association to the replication complex.

Another important actor to regulate replication fork progression is the F-box protein Dia2. It is known that Dia2 interacts with many replication proteins, such as MCMs and GINS [13,27]. Recently, its function was associated with its ability to form a complex with the modular ubiquitin ligase SCF (Skp1/cullin/F box) [28]. A physical interaction between Dia2 and Mrc1 and Ctf4 was demonstrated and was shown that SCF^Dia2^ destabilizes Mrc1 and Ctf4 in a proteasome-dependent manner [29].

Other key actors in the process of S-phase checkpoint activation are the sensor kinase Mec1 (in a complex with Ddc2) and the effector kinase Rad53 [30-32]. Mrc1 is a substrate for Mec1 and is known to directly interact with Rad53 [33]. At first, in Rad53 independent manner, Mrc1 is Mec1 phosphorylated [34]. Thus, Mrc1 becomes competent to bind Rad53 and predisposes it to Mec1 phosphorylation [35]. The activated Rad53 additionally phosphorylates Mrc1. Although its fundamental function is to bring together Rad53 and Mec1 kinases in order to turn the S-phase checkpoint and to stabilize the replication forks [35-37], *mrc1* deletion mutation is not lethal. When knocked out, its function seems to be taken by the specific checkpoint mediator Rad9 [38]. Nevertheless, stalled forks restart much harder in *mrc1Δ* cells when HU is removed from the media, suggesting a role for Mrc1 to promote stable fork-pausing complex formation and to guarantee recovery after fork arrest [39]. Tourriere and co-workers also demonstrate that in *mrc1Δ* cells, as well as in *tof1Δ* mutants, the S-phase seems to be about 20 min longer, compared to the wild type (WT) yeast cells, most probably as a result of the approximately 40% slower progression of the replication fork. Claspin depletion also affects fork progression rates in human cell lines [40]. Although the rate of fork progression seems to be reduced in *tof1Δ* as well, it is less pronounced than that in *mrc1Δ.* The absence of Tof1 seems to reflect much stronger on the pausing of the replication forks at the rDNA replication fork barrier (RFB), protein-DNA barriers sites at the tRNA promoters and centromeres [39,41]. In contrast, in *tof1Δ* and in *mrc1Δ* yeast cells, fork stalling is significantly increased at inverted repeat (IR) provoked hairpins than in WT cells [42]. The authors suggest that both Tof1 and Mrc1 counteract replication fork stalling at such DNA secondary structures.

All the above data indicate the key role of the Tof1/Csm3/Mrc1 complex for normal replication fork movement and the establishment and regulation of the S-phase checkpoint. Our study examines the importance of every protein of Tof1/Csm3/Mrc1 complex for the nuclear localization and consequent chromatin binding of the other two. The specific role of Mrc1 in the process of adaptation to reduced nucleotide levels is also studied.

## Results

### Independent nuclear localization of the subunits of the Tof1/Csm3/Mrc1 complex

As our study aims to examine the interdependences of the *S. cerevisiae* S-phase checkpoint proteins Tof1, Csm3 and Mrc1 with regard to their nuclear localization, we first carried out sequence analysis of those proteins for predictive Nuclear Localization Signals (NLS). The NLS is a sequence on the surface of a protein that is used to target the protein to enter the cell nucleus. We used the ‘PredictNLS’ software [43] that is located at https://rostlab.org/owiki/index.php/PredictNLS. The analysis of the three examined proteins revealed that only Tof1 possesses hypothetical NLS (KKDKRKRRK), starting at the 1013th amino acid. According to the program, this NLS is common for 28 proteins from various organisms, all of them located in nucleus. This prediction data suggest that Tof1 might be the leading protein to target the complex into the nucleus.

As Tof1 is the only one of the three that possesses canonical NLS, we checked whether it is the protein that is responsible for the nuclear localization of Csm3 and consequently Mrc1 in *S. cerevisiae*. To do that, we used GFP-tagged proteins [Invitrogen™; [44]], shortly named *TOF1-GFP*, *CSM3-GFP* and *MRC1-GFP* (See Methods).

**Figure 1:**
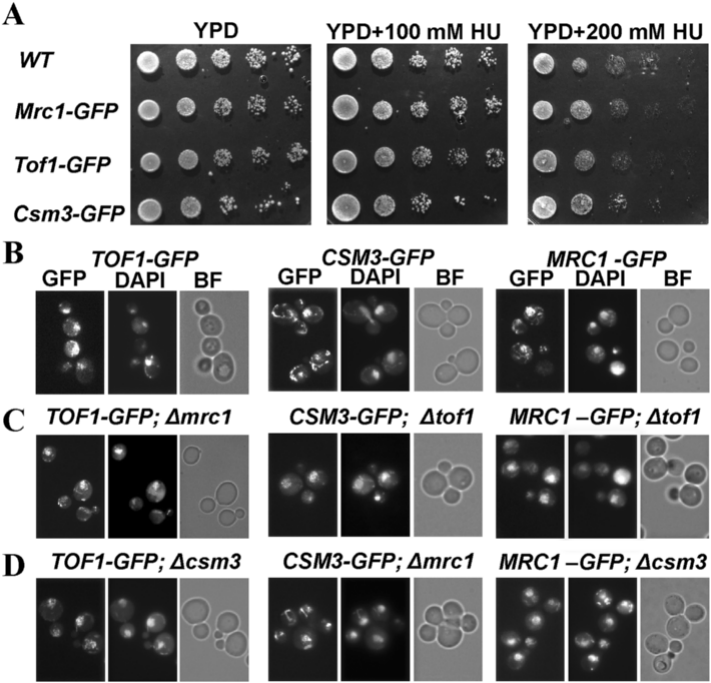
**Independent nuclear localization of the subunits of the Tof1/Csm3/Mrc1 complex. (A)** Viability test of the S-phase checkpoint GFP-tagged proteins by 10-fold serial dilution assay. 5 μL of each dilution are spotted onto YPD, YPD supplemented with 100 mM and 200 mM HU. **(B-D)** All GFP strains are paraformaldehyde fixed and subjected to fluorescent microscopy analysis to detect the position of GFP-tagged proteins in the cell. 2.5 μg/ml DAPI staining is used for all of the probes to visualize the position of nucleus. The obtained GFP and DAPI signals are analyzed for co-localizations. GFP - Filter set 38HE (Zeiss); DAPI - Filter set 01 (Zeiss); BF – bright field.

To make sure that the three S-phase checkpoint GFP-tagged proteins are fully functional, we compared their viability with that of the untagged versions. When grown on a rich YPD media, all three GFP-tagged strands revealed viability comparable to the wild-type control (Figure 1A). The GFP-tagged strands also revealed ability to withstand chronic exposure to two different concentrations of the S-phase checkpoint inducing agent HU, similar to that of the wild-type cells (Figure 1A). Then, assuming that the three GFP-tagged strains function as their untagged versions, we used them to delete a gene coding a partner subunit of the S-phase checkpoint complex Tof1/Csm3/Mrc1 that is not GFP-tagged (See Table 1, Methods). As a result, a full set of deletion mutants of the complex’s subunits was achieved. We will refer to those strains as: *TOF1-GFP; csm3∆*, *TOF1-GFP; mrc1∆*, *CSM3-GFP; tof1∆*, *CSM3-GFP; mrc1∆*, *MRC1-GFP; tof1∆* and *MRC1-GFP; csm3∆*. Asynchronous, exponentially growing cells from the constructed strains, as well as the initial GFP strains without deletions, were paraformaldehyde fixed and subjected to fluorescent microscopy analysis to detect the position of GFP-tagged proteins in the cell. DAPI DNA staining was used for all of the probes to visualize the position of nucleus. Data were documented and analyzed for all of the examined strains (Figure 1B, C, D). As was expected, the control *TOF1-GFP*, *CSM3-GFP* and *MRC1-GFP* strains revealed co-localization of DAPI and GFP signals, indicative of nuclear localization of the respective subunit. Interestingly, all other strains - *TOF1-GFP; csm3∆*, *TOF1-GFP; mrc1∆*, *CSM3-GFP; tof1 ∆*, *CSM3-GFP; mrc1∆*, *MRC1-GFP; tof1∆* and *MRC1-GFP; csm3∆*, also revealed co-localization of their GFP and DAPI signals (Figure 1), suggestive of nuclear localization of the three subunits, regardless of the lack of their partners. Additionally, neither of the studied strains with deletions revealed cytoplasmic accumulation of a GFP-tagged protein. These results demonstrate that in *S. cerevisiae* the three subunits of the Tof1/Csm3/Mrc1 S-phase checkpoint complex are independent with regard to their nuclear translocation.

**Table 1:** **List of *S. cerevisiae *** **strains used in this study**

***Strains (ORF name)***	***Genotype***	***Source***
*TOF1-GFP* (YNL273W)	*MATa his3Δ1 leu2Δ0 met15Δ0 ura3Δ0 tof1-GFP*::His3MX6	Invitrogen™
*MRC1-GFP* (YCL061C)	*MAT*a *his3Δ1 leu2Δ0 met15Δ0 ura3Δ0 mrc1-GFP*-*HIS3MX6*	Invitrogen™
*CSM3-GFP* (YMR048W)	*MAT*a *his3Δ1 leu2Δ0 met15Δ0 ura3Δ0 csm3-GFP*-*HIS3MX6*	Invitrogen™
*RAD53-GFP (YPL153C)*	*MATa his3Δ1 leu2Δ0 met15Δ0 ura3Δ0 rad53-GFP-HIS3MX6*	Invitrogen™
*TOF1-GFP; csm3Δ*	*MAT*a *his3Δ1 leu2Δ0 met15Δ0 ura3Δ0 tof1-GFP*-*HIS3MX6 csm3Δ*::*KanMX*	This study
*TOF1-GFP; mrc1Δ*	*MAT*a *his3Δ1 leu2Δ0 met15Δ0 ura3Δ0 tof1-GFP*-*HIS3MX6 mrc1Δ*::*KanMX*	This study
*CSM3-GFP; tof1Δ*	*MAT*a *his3Δ1 leu2Δ0 met15Δ0 ura3Δ0 csm3-GFP*-*His3MX6 tof1Δ*::*KanMX*	This study
*CSM3-GFP; mrc1Δ*	*MAT*a *his3Δ1 leu2Δ0 met15Δ0 ura3Δ0 csm3-GFP*-*HIS3MX6 mrc1Δ*::*KanMX*	This study
*MRC1-GFP; csm3Δ*	*MAT*a *his3Δ1 leu2Δ0 met15Δ0 ura3Δ0 mrc1-GFP*-*HIS3MX6 csm3Δ*::*KanMX*	This study
*MRC1-GFP; tof1Δ*	*MAT*a *his3Δ1 leu2Δ0 met15Δ0 ura3Δ0 mrc1-GFP*-*HIS3MX6 tof1Δ*::*KanMX*	This study
*HTB2-mCherry*	SLJ3517 (*MATα, htb2::HTB2-mCherry-HYGMX*)	Sue L. Jaspersen

### Interdependence of the subunits of the Tof1/Csm3/Mrc1 complex for their chromatin binding

**Figure 2:**
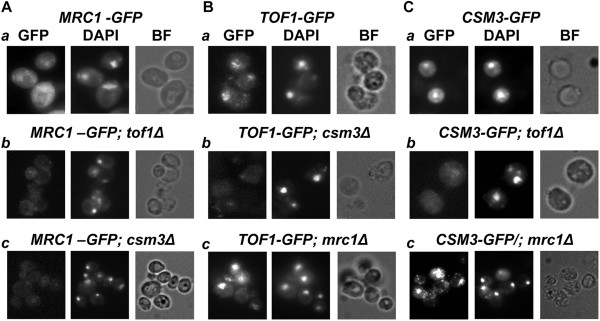
**Interdependence of the subunits of the Tof1/Csm3/Mrc1 complex for their chromatin binding.** After partial spheroplasting by LongLife™ Zymolyase®, all GFP strains are subjected to soluble proteins washing via TritonX-100 detergent treatment. In all experiments where chromatin binding of Mrc1 is studied, cells are treated with 0.5% w/v of detergent. When Tof1 or Csm3 are studied, 3.0% w/v of detergent is used. Cells are paraformaldehyde fixed and subjected to fluorescent microscopy analysis to detect the position of GFP-tagged proteins. 2.5 μg/ml DAPI staining is used for all of the probes to visualize the position of DNA. The obtained GFP and DAPI signals are analyzed for co-localizations. The match of obtained DAPI and GFP signals of TritonX-100 treated *MRC1-GFP***(A.a)**, *TOF1-GFP***(B.a)** and *CSM3-GFP***(C.a)** is indicative of their chromatin bound state. When *csm3* or *tof1* genes are deleted (*TOF1-GFP; csm3∆***(B.b)** and *CSM3-GFP; tof1∆***(C.b)**), the reciprocal binding partner is not detected on chromatin. After TritonX-100 wash of the soluble proteins from *TOF1-GFP; mrc1∆***(B.c)** and *CSM3-GFP; mrc1∆***(C.c)** strains, the GFP signals coincide with DAPI signals. Both *MRC1-GFP; tof1∆***(A.b)** and *MRC1-GFP; csm3∆***(A.c)** strains, when treated with TritonX-100, demonstrate lack of GFP signal. GFP - Filter set 38HE (Zeiss); DAPI - Filter set 01 (Zeiss); BF – bright field.

Next we studied the interrelations of the three subunits for the chromatin assembly of the Tof1/Csm3/Mrc1 complex. In order to develop a whole cell study that can permit direct visualization of chromatin attached proteins, we used the same set of strains and carried out a “soft wash” by TritonX-100 detergent of partially spheroplasted *S.cerevisieae* cells (Methods). The most important step in that procedure was to determine the percentage of detergent to use. First we treated the cells with 100 mM HU for 3 hours to ensure that the three studied proteins are chromatin bound. Then we tested various amounts of TritonX-100 on every GFP-strain without deletion. The maximum percentage of detergent that does not detach the protein from chromatin and reveal nuclear GFP signal was used for further studies. For *MRC1-GFP*, 0.5% w/v of detergent proved to be appropriate and for *TOF1-GFP* and *CSM3-GFP* – 3.0%. w/v (Figure 2). As a positive control, a strain, carrying HTB2 protein (*S. cerevisiae* histone H2B) fused with mCherry was also subjected to the same TritonX-100 washing procedure (Additional file 1: Figure S1). This method permitted us to perform simple multichannel fluorescent microscopy and direct visualization of the insoluble, chromatin bound GFP-tagged proteins. DAPI staining of the paraformaldehyde fixed cells was carried out. The match of GFP and DAPI signals was analyzed as an indicator of chromatin binding of the GFP-fused protein. As the treatment with TritonX-100 of partially spheroplasted yeast cells leads to cell shape deformations, the compactness of the achieved DAPI signal was also representative of nuclear integrity.

The microscopy revealed that when *tof1* or *csm3* genes are deleted (in *CSM3-GFP; tof1∆* or *TOF1-GFP; csm3∆* strains respectively), the reciprocal binding partner was not attached to chromatin (Figure 2), showing that Tof1 and Csm3 are interdependent for their chromatin binding. In contrast, such a dependence of Tof1 and Csm3 on Mrc1 was not observed. After TritonX-100 wash of the soluble proteins and fluorescent microscopy of the *TOF1-GFP; mrc1∆* and *CSM3-GFP; mrc1∆* strains, the examined GFP signals coincided with the corresponding DAPI signals (Figure 2). These results show independence of Tof1-Csm3 dimer chromatin binding on Mrc1. In contrast, Mrc1 required intact Tof1-Csm3 complex in order to associate to chromatin (Figure 2).

### Mrc1 is positioned in the nucleus throughout the cell cycle

**Figure 3:**
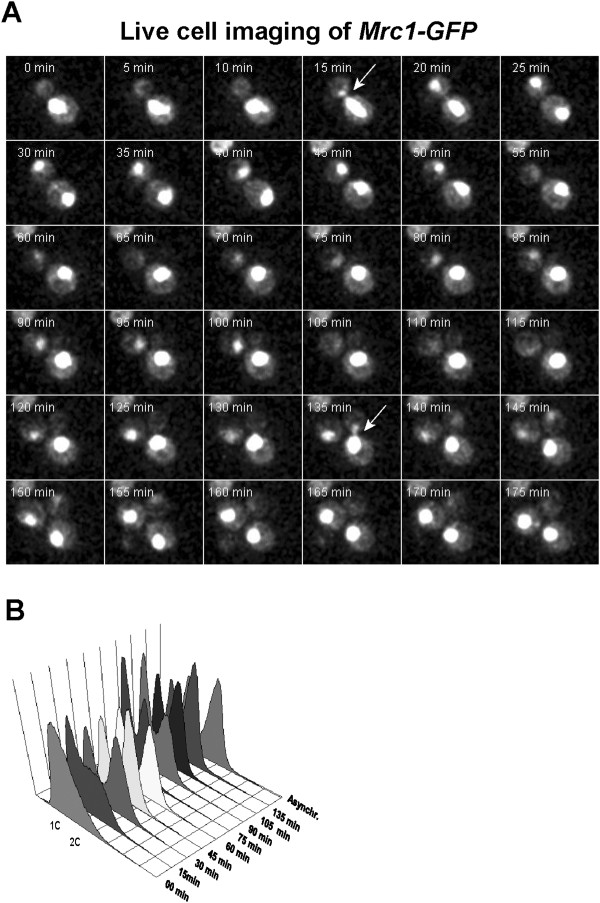
**Nuclear presence of Mrc1 throughout the cell cycle. (A)** Time-lapse live cell imaging of *MRC1-GFP* yeast cell throughout the cell cycle. The arrows indicate the characteristic anaphase nuclear morphology of the cell. The frames situated between two arrows encompass of one entire cell cycle. **(B)** FACS analysis of *MRC1-GFP* yeast cells after release from 100 mM HU arrest.

The three studied proteins co-precipitate together. Interestingly, Tof1 and Csm3 co-precipitate in stoichiometric amounts, but Mrc1 (as well as MCM’s complex subunits) is in substoichiometric amounts [7], suggesting that Mrc1 is not constantly attached to Tof1-Csm3 dimer. As the function of Mrc1 is assumed to be restricted to S-phase, when it is DNA bound [33], we checked whether it is positioned in cell nucleus during that phase of the cell cycle only. To check this possibility, we examined the nuclear localization of Mrc1 during the cell cycle. *S. cerevisiae* exponentially growing *MRC1-GFP* cells were subjected to time-lapse live cell imaging. The obtained results indicated that Mrc1 fluorescent signal is detected in the cell nucleus during the entire cell cycle (Figure 3A, B). These data show that translocation into the nucleus is not the key process to restrict the attachement of Mrc1 to Tof1-Csm3. Probably, some other mechanisms, such as protein-protein and/or protein DNA interactions, as well as degradation of Mrc1 are the responsible mechanisms, which control its complex binding and S-phase functions.

### Mrc1 is removed from chromatin when the S-phase checkpoint is overcome in the presence of HU

Tof1, Csm3 and Mrc1 are all necessary for normal fork progression as well as stable checkpoint fork arrest [8,45], but just Mrc1 is sufficient to guarantee recovery after fork arrest [46]. It was shown that replication forks reveal restart difficulties after HU block in *mrc1∆* cells [39]. As Mrc1 is important for stable fork arrest, we decided to check for alterations in the nuclear behavior of Mrc1 in the process of adaptation. The adaptation is a process of overcoming the S-phase checkpoint and is noticed to take place when cells are subjected to persistent agent treatment or impossibility to repair specific DNA damage.

**Figure 4:**
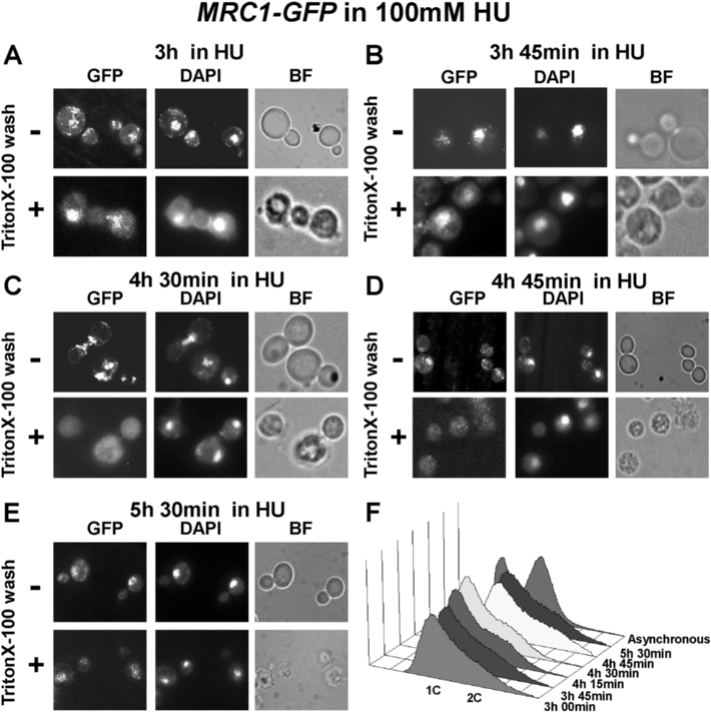
**Alterations in the nuclear behavior of Mrc1 during the process of adaptation. (A-E)***MRC1-GFP* probes from indicated tome points, taken in the presence of 100 mM HU. **(-)** Paraformaldehyde fixed and DAPI stained cells, subjected to multichannel fluorescent microscopy, not washed with detergent. **(+)** TritonX-100 washed and consequently fixed and DAPI stained probes, observed under fluorescent microscope. GFP - Filter set 38HE (Zeiss); DAPI - Filter set 01 (Zeiss); BF – bright field. **(F)** FACS analysis of the studied time points in 100 mM HU, indicating a shift towards 2C after 4 h and 30 min.

**Figure 5:**
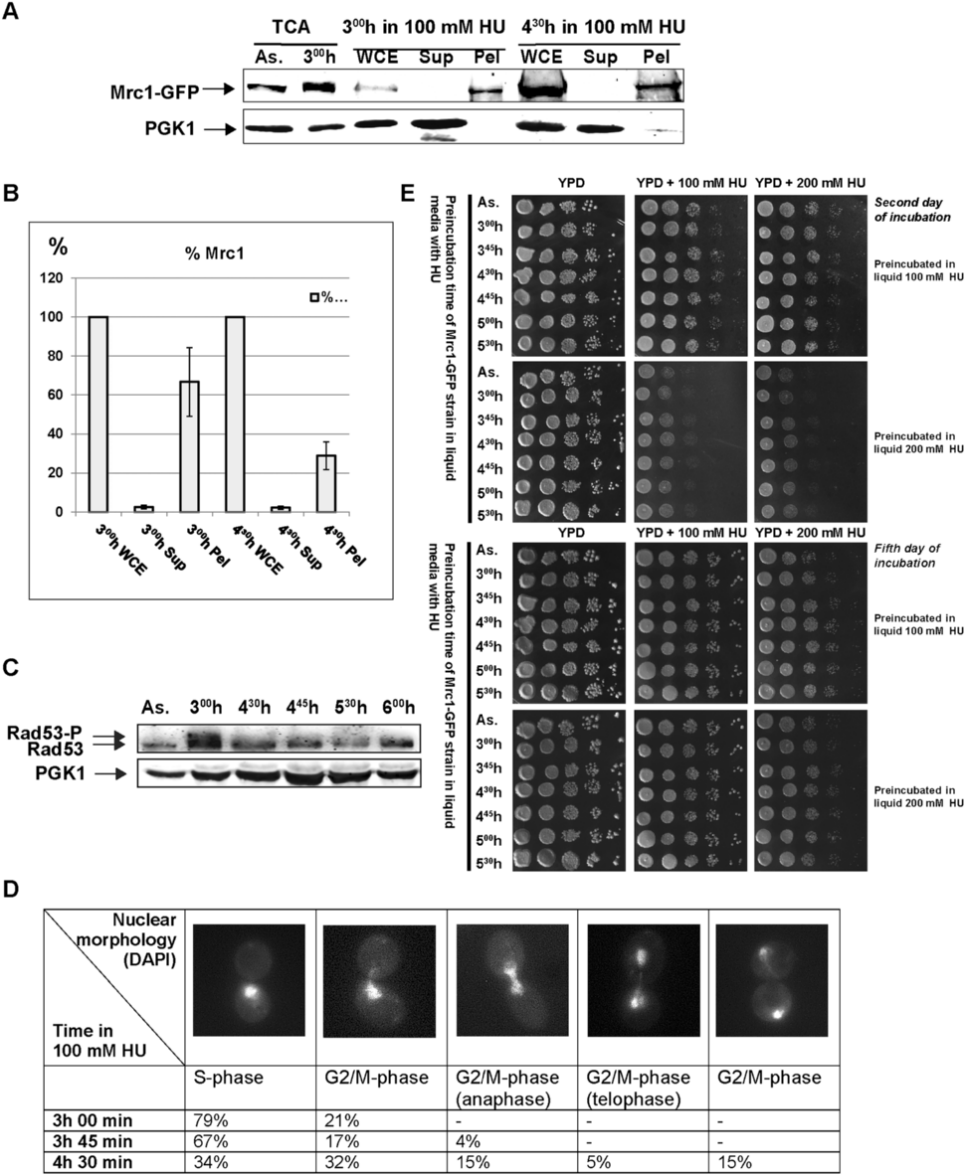
***Mrc1-GFP *cells by-pass the S-phase checkpoint. (A)** Bulk chromatin fractioning assay of *Mrc1-GFP* strain in the presence of 100 mM HU. Samples from indicated time points were fractionated into whole cell extract (WCE), crude soluble (Sup) and chromatin (Pel) fractions. Yeast total protein extracts (TCA) from asynchronous (As.) and treated for three hours (3^00^ h) with 100 mM HU *MRC1-GFP* cells were run on the same 6–15% SDS-PAGE. As a control, the cytoplasmic PGK1 protein was monitored on the same Western blotting membrane. **(B)** The average amount of Mrc1-GFP protein from different Bulk chromatin fractioning assays, measured by the Gel analysis tool of ImageJ software. The measured amount of Mrc1-GFP from the WCE of each time point is assumed as 100%, the protein from Sup and Pel is calculated as a percentage of respective WCE. Standard deviation of means is indicated as error bars. **(C)** Total protein extracts from indicated time points from *Mrc1-GFP* cells in the presence of 100 mM HU. Samples were run on a 6–15% SDS-PAGE and after western blotting, immunodetections of Rad53 and PGK1 (loading control) were carried out. Rad53-P and Rad53 indicate the phosphorylated and unphosphorylated forms of Rad53 protein. **(D)***Mrc1-GFP* strain nuclear morphology analysis of treated with 100 mM HU yeast cells. Samples from indicated time points were DAPI stained and monitored under fluorescent microscope. The number of cells with indicated nuclear morphology is given as a percentage of the sum of all counted cells from each sample. **(E)** Exponentially growing *S. cerevisiae* cells from *MRC1-GFP* strain were arrested with 100 mM HU or 200 mM HU for 3 h in liquid YPD medium and then plated on YPD and YPD, containing 100 or 200 mM HU respectively at indicated time points. The duration of incubations is indicated.

*MRC1-GFP* cells were treated with 100 mM HU. Samples, starting from the third hour, from indicated time points, were taken and either fixed straight away or washed with TritonX-100 before fixing. The fluorescent microscopy results indicated the presence of Mrc1 at chromatin until 3 h 45 min (Figure 4A, B). Interestingly, the next samples, taken at 4 h 30 min and 4 h 45 min (Figure 4C, D), indicated that Mrc1 was still located in cell nucleus (although its amount seemed to be diminished) but removed from chromatin. Then, after 5 h 30 min from HU addition (Figure 4E), Mrc1 protein seemed to reappear at chromatin. The flow cytometry analysis, carried out with cell probes from the same time points, indicated a small shift towards two contents of DNA (Figure 4F) after 4 h 30 min, suggesting that the S-phase checkpoint arrest had been by-passed and that the yeast cells had overcome the ribonucleotide reductase inhibition.The observed Mrc1 chromatin diminishment was also detected by bulk chromatin fractioning assay (Figure 5A). The Western blot band analysis of that experiment revealed that the amount of chromatin bound Mrc1-GFP protein from 4 h 30 min time point, relative to the protein value from the whole cell extract of the same time-point, indicated 42% diminishment, compared to the same correlation from the 3 h 00 min in 100 mM HU (Figure 5B).

To ensure that cells that proceed throughout the cell cycle in the presence of HU had overcome the S-phase checkpoint arrest, we analyzed the Rad53 kinase. We carried out Western blot analysis of total protein extracts from 100 mM HU treated yeast *MRC1-GFP* cells (Figure 4G). The immunodetection indicated (Figure 5C) that as expected, at the third hour Rad53 appeared to be in hyperphosphorylated state that corresponds to S-phase checkpoint activation. The samples taken later on show that the amount of phosphorylated Rad53 seemed to significantly decrease. Both detachment of Mrc1 from chromatin and the decrease of phosphorylated Rad53 are indicative for S-phase checkpoint escape.

Confirmatory of that S-phase checkpoint escape are the results we obtained from *Mrc1-GFP* yeast nuclear morphology analysis experiment [47]. Yeast cells, treated with 100 mM HU as described were DAPI stained and monitored under fluorescent microscope at indicated time points (Figure 5D). At the time of HU arrest, 79% of the counted cells revealed S-phase characteristic cellular and nuclear morphology. In contrast, at 4 h and 30 min time point, that percentage dropped to 34%. This shows that 45% of the cells had exited the S-phase, a value close to the calculated 42% decrease of chromatin bound Mrc1. The rest of the cells indicated G2/M-phase related morphology, indicating that cells were no longer S-phase arrested and are trying to continue the cell cycle (Figure 5D). In addition, to visualize the behavior of studied proteins during S-phase checkpoint escape on a single cell level, we carried out time-lapse live cell microscopy of *Mrc1-GFP* and *Rad53-GFP* strains in the presence of 100 mM HU (Additional file 1: Figure S2B and C and Additional file 2: Movie S2 and Additional file 3: Movie S4). The results from those experiments confirmed that cells continue their cell cycle progression in the presence of HU, but the duration of the cell cycle was much longer (compare Additional file 1: Figure S2A with S2B and Figure S2C with Figure 3). Movies of all time-lapse experiments are available in the (Additional file 2: Movie S2, Additional file 3: Movie S4, Additional file 4: Movie S1 and Additional file 5: Movie S3).

To check whether cells can steadily surmount the HU provoked nucleotide deficiency, a HU-viability test was carried out. Samples from *MRC1-GFP* cells, preliminarily arrested with HU (100 mM HU and 200 mM HU), were taken at indicated time points and grown on plates with or without HU (100 or 200 mM respectively). All samples indicated a visible cell growth (Figure 5E). Not surprisingly, a difference in growth was observed between cells, which after HU preincubation were plated on YPD containing HU and those – on plates without HU (best visualized after two days of incubation – Figure 5E). The results show that all samples, even those, incubated on plates containing 200 mM HU, continued to grow, demonstrating that cells had surmounted the new conditions.

## Discussion

Our study aimed to estimate the interdependencies of the three proteins with regard to compartmentalization of complex assembly. Recent data from other eukaryotes lead to the idea that Tof1/Csm3/Mrc1 complex is assembled in cell cytoplasm. Tanaka and co-workers demonstrated in *S. pombe* that the amount of Mrc1-GFP nuclear signal is significantly reduced when Swi1 or Swi3 is deleted [48]. The authors suggest that the Swi1 and Swi3 are important for Mrc1 nuclear localization and consequent DNA binding. Their results presume that a dimer of Tim-Tipin is assembled in cytoplasm and is responsible for the nuclear translocation of Mrc1. In HeLa cells, when Tim or Tipin were knocked-down, the respective binding partner was relocated to cytoplasm. In addition, the amount of that partner was significantly reduced [49]. Such dependence for Claspins’s amount was not found for asynchronous growing cells. But when Tim or Tipin siRNA treated cells were subjected to HU, the amount of nuclear Claspin seemed to diminish. The authors suggest that Tim and Tipin facilitate Claspin nuclear localization under replication stress.

To check whether the complex is formed in the cytoplasm in *S. cerevisiae*, we studied the cellular localization of GFP-tagged subunits of Tof1/Csm3/Mrc1, when the complex is intact and when a subunit is missing. As in higher eukaryotes [49], our analysis revealed a hypothetical NLS for Tof1, suggesting its responsibility for the nuclear translocation of the other subunits of the complex. However, in contrast to higher eukaryotes, independence with regard to nuclear translocation of all three subunits was observed. Our findings demonstrate that in contrast to *S. pombe* and human cells, the *S. cerevisiae* Tof1/Csm3/Mrc1 S-phase checkpoint complex is most probably assembled in cell nucleus, as every subunit can enter it independently of the others. Deletion of each of the three genes did not lead to cytoplasmic accumulation of any partner subunit. The amounts of the undeleted, GFP-tagged proteins also seemed to be unaffected when observed under fluorescent microscope. As budding yeast is a preferred model organism for replication studies, such a difference in proteins relationships and cell positioning control must be taken into account. As yeast cell nucleus is not disassembled during cell division, but undergoes nuclear division into two daughter nuclei, it might be speculated that those differences in the localization of complex assembly are a result of evolutionary adaptation and management of higher eukaryotes.

As Tof1, Csm3 and Mrc1 enter nucleus independently in *S. cerevisiae,* a question about the mechanism of complex assembly arises. The stoichiometric interaction of Tof1 and Csm3 (in co-precipitation experiments on asynchronous cultures) indicates that by default they form a heterodimer (Nedelcheva et al. [7]). In contrast, the non- stoichiometric amount of Mrc1 suggests that it joins the complex occasionally. Is then the Tof1-Csm3 dimer formation required to ensure attachment to chromatin and how is Mrc1 related to those relationships? In higher eukaryotes data are variable in accordance with the model system applied in the study. The *Xenopus* egg extract data show that Tipin and Claspin fail to bind to chromatin when Tim1 is depleted and that Tim1-Tipin is required for binding of Claspin but not vice versa [50]. In HeLa cells Tim and Tipin are interdependent for their chromatin binding [49], but insoluble Claspin is not affected by Tipin siRNA in asynchronous culture. Such dependence is noticed in HU treated cells only.

To visualize the chromatin association and dependencies of the subunits via a whole cell approach, we adapted the higher eukaryotes protocol for *in situ* chromatin binding assay [36,37] to *S. cerevisiae*. This method permits performance of direct multichannel fluorescent microscopy to visualize the insoluble, chromatin bound GFP-tagged proteins. The results demonstrate the necessity of Tof1 for the chromatin binding of Csm3 and vice versa. Our results also show that Mrc1 is Tof1 and Csm3 dependent for its chromatin association, but in contrast, Mrc1 is not required for the chromatin binding of Tof1 and Csm3. These data are in unison with ChIP-chip results of Bando and co-workers for the co-dependences of those proteins for association with replication forks [1].

It can be summarised that in *S. cerevisiae* Tof1-Csm3 initial dimer formation is required for chromatin association. The nuclear import is not a regulatory step as every single subunit enters the nucleus independently. As a dimer Tof1-Csm3 is responsible for the chromatin binding of Mrc1.

A logical question to answer in that relation is whether the controlling mechanism for the restricted binding of Mrc1 to the Tof1 - Csm3 dimer (as co-precipitations indicated) is a result of cell cycle oscillations of its cellular localization. As Mrc1 known functions are restrained to S-phase of the cell cycle, when it is DNA bound [33], we checked whether it is positioned in cell nucleus during that phase of the cell cycle only. Our results indicated that there is nuclear Mrc1 during the entire cell cycle, confirming that translocation into the nucleus is not the leading process to regulate the association of Mrc1 to the S-phase checkpoint complex. A regulatory function of the timing of Mrc1’s triple S-phase complex association can be suggested, but further studies are required to elucidate the fine mechanisms of these interactions.

All three proteins Tof1, Csm3 and Mrc1 are involved in normal fork progression and stable checkpoint fork arrest [8,45], but just Mrc1 is assumed to be responsible for fork rehabilitation after fork arrest [39]. Stalled forks restart much harder in *mrc1Δ* cells when HU is removed from the media, suggesting a role for Mrc1 to promote stable fork-pausing complex formation and to guarantee recovery after fork arrest. Some recent data connect Mrc1 with SCF^Dia2^. This interaction was shown to be responsible for destabilization of Mrc1 in a proteasome-dependent manner during S-phase of the cell cycle in vivo [29]. It was also demonstrated that Dia2 contributes to Mrc1 degradation during S-phase checkpoint recovery [46]. As Mrc1 is important for stable fork arrest, we checked for alterations in the nuclear behavior of the protein in the process of overcoming the S-phase checkpoint, called adaptation. Generally, the adaptation is a process of loosening the S-phase checkpoint when cell meets impediments for coping with persistent agent or damage. The reason for such cell decision is not quite clear. It was suggested that when the cell is unable to cope with the problem, it allows restoration of the cell cycle to provide opportunities to repair the damage in a subsequent cell cycle, enhancing its chances for survival. The intimate mechanisms of executing and controlling this phenomenon are still vague. Some data point out the specific role of the amount of polo-like kinase CDC5 to suppress the hyperphosphorylation of Rad53 that leads to relieve of cell division arrest [51,52].

We studied the nuclear behavior of Mrc1 after a prolonged period of incubation in the presence of HU. The fluorescent microscopy results in combination with flow cytometry data and bulk chromatin fractioning indicated that when the *S. cerevisiae* cell by-passes S-phase checkpoint arrest (after 4 h and 30 min) to proceed further into the cell cycle in the presence of the blocking agent, Mrc1 dissociates from chromatin. This finding emphasizes the specific role of Mrc1 for keeping the stability of forks arrest. It shows that the physical presence of Mrc1 at replication pausing complex is required not only for stable fork arrest in response to S-phase checkpoint agent, but for the duration of that arrest as well.

In support of our findings, cell free studies on *Xenopus* egg extract in aphidicolin-induced DNA replication block show that after a prolonged interphase arrest, the extracts undergo adaptation and enter into mitosis with unfinished DNA replication. In this process Chk1 undergoes inactivation and Claspin dissociates from chromatin [53].

**Figure 6:**
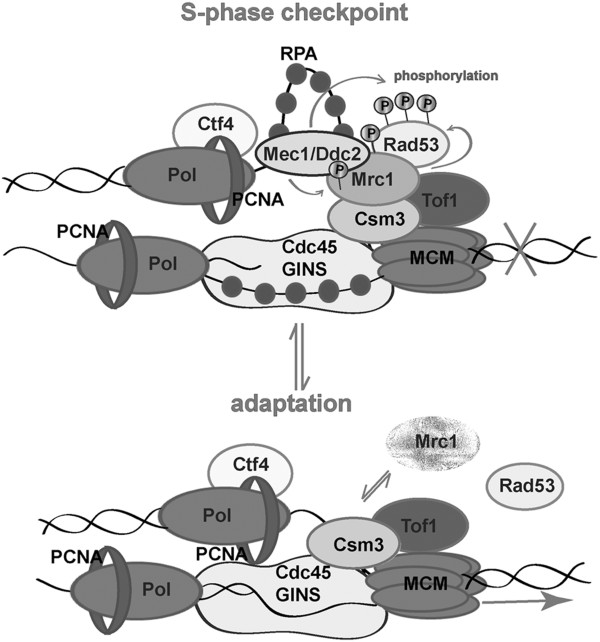
The role of Mrc1 for the duration of the S-phase checkpoint arrest.

One of the major functions of the S-phase checkpoint is to sufficiently enlarge the nucleotide pool in the cell [54]. The key enzyme to regulate the levels of dNTPs – ribonucleotide reductase (RNR) is regulated by the Mec1/Rad53/Dun1 kinases via two different mechanisms [55,56]. The first one aims the transcriptional induction of the RNR genes and the second results in phosphorylation and removal of the RNR inhibitor Sml1 [57,58]. When HU is introduced into the media, it provokes the S-phase checkpoint activation that results in Sml1 degradation and free nucleotide pool enlargement. On the other hand, the HU itself is an inhibitor of RNR and effects limitation of the amount of dNTPs. Therefore, the net effect of the two counteractive processes is measured and the predominant process takes control over cell fate, directing it either towards nucleotide synthesis or towards nucleotide synthesis inhibition. The dose of HU itself might be of major importance to target the process. In our experiments 100 mM HU was used. Our FACS analysis (Figures 3B and 4F) and the phosphorylation of Rad53 at the third hour of HU treatment (Figure 5C) indicate that the amount of HU used provokes S-phase checkpoint activation. At the same time, the live-cell imaging of Rad53-GFP and Mrc1-GFP cells (Additional file 1: Figure S2), as well as the viability test that we carried out (Figure 5E) undoubtedly indicate that yeast cells somehow succeed to survive and grow in 100 and even 200 mM HU for a long period of time. This shows that the decision for cell arrest was abolished and probably the nucleotide levels were sufficiently adequate to allow cell cycle restoration. Interestingly, the ordinary dynamics of the cell cycle is not absolutely restored. Alvino and co-workers [59] as well as our time-lapse live cell imaging experiments indicate that the duration of the cell cycle in HU is much longer than the ordinary one. A probable explanation is that cells adapt to HU by raising the level of nucleotides to permit progression of the cell cycle, but those levels remain limited and do not allow full restoration of the dynamics of the cell cycle. How the cell weighs the two opposite effects of the HU on the dNTPs pool and takes its decisions is still unclear. Many other experimental data are required to establish the mechanisms of this decision making. Our results indicate that the detachment of Mrc1 from chromatin and the diminishment of Rad53 phosphorylation give a proof of S-phase checkpoint bypass that follows that adaptation.Our results demonstrate that in the living cell, during S-phase checkpoint adaptation, Mrc1 is removed from chromatin. One possible explanation for that detachment is the specific role of Mrc1 to prevent replicative helicase movement when the polymerase meets an obstacle for correct DNA synthesis. We hypothesize that the pausing structure is possible when a “clutch” of Mrc1 on MCM is present. This clutch is required for docking Mec1and Rad53 to ensure their checkpoint kinase activities, leading to fork arrest. Via some regulatory mechanisms (perhaps by means of Polo-like kinase attachment and consequent phosphorylation) Mrc1 is dissociated from chromatin by detachment or degradation. The lack of Mrc1 leads to loss of Rad53/Mrc1 activity and loosens MCM helicase (Figure 6). As Mrc1 is required for normal replication fork progression, later on, when the cell division arrest has been relieved, the protein rebinds Tof1-Csm3 to ensure DNA synthesis.

## Conclusions

Our findings demonstrate that in contrast to *S. pombe* and human cells, the *S. cerevisiae* Tof1/Csm3/Mrc1 S-phase checkpoint complex is most probably assembled in cell nucleus, as every subunit can enter it independently and deletion of each of the three genes did not lead to cytoplasmic accumulation of any partner subunit.

Our data also indicates that in *S. cerevisiae* Tof1-Csm3 initial dimer formation is required for chromatin association of Mrc1. This process is not controlled by the cell cycle as the protein is constantly positioned in the nucleus.

Our results indicate that in the process of adaptation to the presence of HU Mrc1 is detached from chromatin to relax Rad53 activity and thus to allow completion of the cell cycle. Our study emphasizes the specific role of Mrc1 for keeping the stability of fork arrest. It shows that the physical presence of Mrc1 at replication pausing complex is required not only for stable fork arrest in response to S-phase checkpoint agent, but also for the duration of that arrest.

## Methods

### Strains and media

*S. cerevisiae* strains (Invitrogen™, Cat.# 95702) YNL273W (*MAT***a***his3Δ1 leu2Δ0 met15Δ0 ura3Δ0 TOF1-GFP-HIS3MX6*), YCL061C (*MAT***a***his3Δ1 leu2Δ0 met15Δ0 ura3Δ0 MRC1-GFP-His3MX6*), YMR048W (*MAT***a***his3Δ1 leu2Δ0 met15Δ0 ura3Δ0 CSM3-GFP-His3MX6*) and YPL153C (*MAT***a***his3Δ1 leu2Δ0 met15Δ0 ura3Δ0 RAD53-GFP-HIS3MX6*) are used [44]. We refer to those strains as *TOF1-GFP*, *MRC1-GFP*, *CSM3-GFP* and *RAD53-GFP*, respectively. All are with BY 4741 background. We used YNL273W, YCL061C and YMR048W to delete a gene coding a partner subunit of the S-phase checkpoint complex Tof1/Csm3/Mrc1 that is not GFP-tagged. As a control for in situ chromatin binding assays SLJ3517 (*MAT* ***α****, htb2::HTB2-mCherry-HYGMX*) or shortly - *HTB2-mCherry*, was used. All strains are listed in Table 1. Strains were cultivated in YPD medium (1% (w/v) yeast extract (Difco), 2% (w/v) Bacto peptone (Difco), 2% (w/v) dextrose). Before fluorescent and confocal microscopy procedures, yeast cells were grown in minimal medium (1.7 g/l YNB, 0.04 g/l CSM-His (Bio101, Inc.) and 2% (w/v) dextrose) to diminish the autofluorescence.

### Construction of strains

The plasmid pKS-KanMX6-1 (4571 bp) was used to PCR amplify the disruption cassettes for *tof1*, *mrc1* and *csm3*. For construction of the disruption cassettes, primers 1–6 were used (Table 2). These cassettes contained a selection marker KanMX for geneticine (G418) resistance. The disruption cassettes carried 50 bp flanking sequences (introduced by the PCR primers) homologous to regions, which surround the target genes. Strains *TOF1-GFP*, *MRC1-GFP* and *CSM3-GFP* were transformed with the respective cassette [60]. The selection for integration was carried out on YPD medium, containing 200 μmol/ml G418. The integration of the disruption cassette was also confirmed by diagnostic PCR [61]. The pairs of primers used in these reactions were designed so that one of them is complementary to the sequence from the KanMX gene, and the other – to the yeast genome region, neighboring the integrated cassette (Table 2).

**Table 2:** PCR primers used in this study

***Primer***	***Aplication***	***Sequence***
scm3 F	Gene disruption	TACTGGATTAAAATGCCATGAAAACGTGAACAGAAACTTTTATTGAGGTCCACTCAACCCTATCTCGG
csm3 R	Gene disruption	TATAGATGCCCACACGCACGTTTGGATTATTACCTTCAATGACATTGCTACTCGAAATTAACCCTCAC
mrc1 F	Gene disruption	CTAAGGAAGTTCGTTATTCGCTTTTGAACTTATCACCAAATATTTTAGTGCACTCAACCCTATCTCGG
mrc1 R	Gene disruption	GACAGCTTCTGGAGTTCAATCAACTTCTTCGGAAAAGATAAAAAACCACTCTCGAAATTAACCCTCAC
tof1 F	Gene disruption	CATCTAGCTTGTGGGGTTTAGTGTATCTTTAATATAGGAGGGCGCACACTCACTCAACCCTATCTCGG
tof1 R	Gene disruption	TTCTAAAATTACACGTATTAAAGGGATTAATTACTACATATTCATTCTCACTCGAAATTAACCCTCAC
KanMX-check	Diagnostic PCR	GTCACCTAAATCGTATGT
csmΔ-check	Diagnostic PCR	ATCGTTTGACAAGAGAGT
mrc1Δ-check	Diagnostic PCR	TCAAATGTCCAAGTGAAC
tof1Δ-check	Diagnostic PCR	GAAGAAGTTACTCCAAGA

### GFP-fixation

A protocol published at “Koshland Web Site/Methods” was applied http://mcb.berkeley.edu/labs/koshland/Protocols/MICROSCOPY/gfpfix.html. 250 μl of *S. cerevisiae* cells were resuspended in 100 μl of paraformaldehyde/sucrose (4 g paraformaldehyde, 3.4 g sucrose in 100 ml water). After 15 min of room temperature incubation, cells were washed and resuspended in appropriate quantity of KPO4/sorbitol (2 M sorbitol, 1 M KPi, pH 7.5 (made of 183.4 ml 1 M K_2_HPO_4_ and 16.6 ml 1 M KH_2_PO_4_) and H_2_O in a 6:1:3 proportion).

### Fluorescent microscopy

Glass slides were covered with 0.1% w/v Poly-L-Lysine solution (SIGMA-ALDRICH, No. P 8920). 2.5 μg/ml of DAPI (SIGMA-ALDRICH, No. 9542) was added to the fixed cells. They were incubated at 30°C, for 15 min in dark. Yeast cells were pelleted and washed in 1×PBS (137 mM NaCl, 2.7 mM KCl, 4.3 mM Na_2_HPO_4_, 1.4 mM KH_2_PO_4_). After resuspending in 1xPBS, they were ready for DNA nuclear observation. 5 μl cell suspension was pipetted onto the slide and after 10 sec deposition, covered with coverslip. Observations are made by EC-Plan Neofluar 100×/1.3 Oil-immersion objective mounted on a Axiovert 200 M inverted fluorescent microscope, Carl Zeiss, using AxioCam MRm CCD camera, Carl Zeiss and filters: Filter set 01 (excitation: BP 365/12; beamsplitter: FT 395; emission: LP 397) and Filter set 38HE (excitation: BP 470/40; beamsplitter: FT 495; emission: BP 525/50). Images were acquired and processed by Carl Zeiss AxioVision Rel.4.7 and ImageJ software.

### Time-lapse live cell imaging experiments

The glass-slide part of the “Glass bottom dishes” (Mattech, #s: P50G-1.5-14-) was covered with 0.1% w/v Poly-L-Lysine solution (SIGMA-ALDRICH, No. P 8920) in order to mount the yeast cells. To diminish the autofluorescence, the yeast cells were grown in minimal medium (1.7 g/l YNB, 0.04 g/l CSM-His (Bio101, Inc. and 2% (w/v) dextrose) with 20 μg/ml extra adenine. All procedures were carried out at 25°C and according to the protocol, described by Silva and co-workers [62] (Cite). Observations were made by CFI Apo TIRF 100X Oil 1.49 NA objective mounted on Yokogawa CSU-X1 spinning disc confocal microscope (Andor Revolution XD system with Nikon TiE microscope stand and incubator for temperature and humidity control). Data were documented by iXon 897EMCCD camera with TiCAM. All time-lapse experiments were run using the following parameters: 11 Z-stacks, 0.5 μm apart, acquired with 18% laser power on 488 nm and 200 ms exposure. Acquisition was made on every 5 min for 12–16 h. Maximum intensity projections of the stacks were prepared using ImageJ. Images and movies were processed by ImageJ software.

### In situ chromatin binding assay

First the procedure was done on *Mrc1-GFP*, *Tof1-GFP* and *Csm3-GFP* strains. Cells were treated with 100 mM HU for 3 hours to ensure that the three GFP-fused proteins are chromatin bound. The cells were washed and resuspended in Spheroplasting buffer (0.1 М КРi_,_ рН 7.5 – described above; 1.2 М sorbitol; 0.5 mM MgCl_2_). 2% of beta-mercapto-ethanol, diluted 1:10, was added and cells were incubated for 7 min at 30°C. 4.0 μl of LongLife™ Zymolyase® (Geno Technology,Inc; Cat. # 786–036) [1.5 U/μl] was added and incubation at 37°C for 13 min was carried out. After centrifugation, spheroplasts were resuspended in Spheroplasting buffer, containing protease inhibitors (Complete Mini EDTA-free tablete, Roche) and various amounts of TritonX-100 were tested for every strain. The maximum percentage of detergent that does not detach the protein from chromatin and reveal nuclear GFP signal was used for further studies. For *MRC1-GFP*, 0.5% w/v of detergent proved to be appropriate and for *TOF1-GFP* and *CSM3-GFP* – 3.0%. w/v. After incubation with TritonX-100 at 20°C for 7 min, cells were washed in Spheroplasting buffer containing protease inhibitors, spun down at 3000 rpm and resuspended in paraformaldehyde/sucrose for GFP-fixation as described above, avoiding vigorous shaking. To study the other *S. cerevisiae* GFP strains with deletions, asynchronous cell cultures were used.

### Spot assays

For 10-fold serial dilutions assays, yeast samples were prepared from exponentially growing cultures with concentration 3.4 × 10^6^ cells/ml. 5 μl of each dilution were then spotted onto YPD and YPD supplemented with 100 mM and 200 mM hydroxyurea (HU). Plates were incubated at 25°C for 3 days.

For HU viability test, exponentially growing *S. cerevisiae* cells from *MRC1-GFP* strain were arrested with 100 mM HU or 200 mM HU in liquid YPD medium. Aliquots, containing 3.4 × 10^6^ cells/ml were taken at indicated time points. 10-fold serial dilutions were prepared and 5 μl of each dilution were spotted onto YPD and YPD, containing 100 or 200 mM HU, respectively. Plates were incubated at 25°C for 5 days.

### Bulk chromatin fractionation

Whole cell, soluble and chromatin fractions were prepared as previously described [63], with modifications. 1 × 10^9^ cells from logarithmic, 100 mM HU treated culture were harvested and 0.1% NaN_3_ was added. After incubation for 5 min at 30°C cells were treated with 3 ml of prespheroplasting buffer [100 mM PIPES (pH 9.4), 10 mM DTT] for 10 min and then in 2 ml spheroplasting buffer [50 mM KH_2_PO_4_/K_2_HPO_4_ (pH 7.5), 0.6 M Sorbitol, 10 mM DTT]. After LongLife™ Zymolyase® (Geno Technology,Inc; Cat. # 786–036) digestion, the spheroplast pellets were washed with 1 ml of ice-cold wash buffer [100 mM KCl, 50 mM HEPES-KOH (pH 7.5), 2.5 mM MgCl_2_, and 0.4 M Sorbitol], pelleted at 4000 rpm for 1 min at 4°C, and resuspended in an equal to the resultant pellet volume of extraction buffer [EB; 100 mM KCl, 50 mM HEPES-KOH (pH 7.5), 2.5 mM MgCl_2_, 50 mM NaF, 5 mM Na_4_P_2_O_7_, 0.1 mM NaVO_3_], containing 1.5% Triton X-100, 1 mM PMSF and protease inhibitors cocktail (cOmplete Mini EDTA-free Protease Inhibitor Cocktail Tablets, # 05892791001, Roche)]. The suspension was divided into two equal parts – one for whole cell extract (WCE) and the second for crude soluble (Sup) and chromatin (Pel) fractions. After 10 min incubation at 4°C, the lysates were passed through a thin syringe needle and spun at 300 × g in order to pellet and discard the aggregated and unlyzed cells. The Sup + Pel fraction lysate was underlayered with 50% volume of 30% sucrose and spun at 12 000 rpm for 10 min at 4°C. The supernatant (Sup) was kept for soluble fraction. Pellet was washed with 25% volume of EB containing 1.5% Triton X-100 (EBX), and spun again at 10 000 rpm for 5 min at 4°C. The crude chromatin pellet was dissolved in EBX. Finally, the volumes of WCE, Sup and Pel were equalized with EBX and 2× Laemmli’s buffer was added to each fraction. Samples were boiled for 3 min, and spun at 10 000 rpm for 1 min before loading to 6-15% gradient SDS PAGE gels.

### Western blotting

Yeast total protein extracts were prepared according to Foiani and co-workers by means of TCA precipitation [64]. All solutions contained protease inhibitors cocktail (cOmplete Mini EDTA-free Protease Inhibitor Cocktail Tablets, # 05892791001, Roche) and the phosphatase inhibitors 0.1 mM Na_3_VO_4_ and 1 mM NaF. The protein aliquots were loaded on 6-15% gradient SDS-PAGE and run on 140 V. The samples were transferred onto Protran nitrocellulose membrane and immunodetected using goat polyclonal anti Rad53 antibody (Rad53 y-19 from Santa Cruz Biotechnology, Santa Cruz, CA). The results were visualized on Odyssey Infrared Imaging system (Li-Cor) by means of IRDye 680RD Donkey Anti-Goat Antibody (# 926–68074, Li-Cor). For detection of Mrc1 protein, mouse monoclonal Anti-GFP Antibody (# 11 814 460 001, Roche) and IR Dye 800CW Goat Anti-Mouse Antobody (#926-32210, Li-Cor) were used. PGK1 was immunodetected by mouse monoclonal anti-PGK1 antibody [22C5D8] (ab113687, Abcam) and IR Dye 800CW Goat Anti-Mouse Antobody (#926-32210, Li-Cor).

## Abbreviations

HU: Hydroxyurea; WT: Wild type; MCM: Minichromosome maintenance proteins; NLS: Nuclear Localization Signals; GFP: Green Fluorescent Protein; DAPI: 4′-6-diamidino-2-phenylindole; ChIP: Chromatin immunoprecipitation; RNR: Ribonucleotide reductase; SDS-PAGE: Sodium Dodecyl Sulfate-Polyacrylamide Gel Electrophoresis.

## Competing interests

The authors declare that they have no competing interests.

## Authors’ contributions

SDU carried out the viability tests, bulk chromatin fractionation, all western blotting experiments and some of the microscopy experiments. ASZ participated in the construction of yeast strains, some of the microscopy experiments and carried out the in situ chromatin binding assays. AMI constructed some of the yeast strains. SSS participated in the design of the study. MNN-V designed the experiments, drafted the manuscript and guided all of the experiments. All authors read and approved the final manuscript.

## Authors’ information

ASZ and AMI were Master degree diploma students, supervised by MNN-V, SDU is a PhD student, supervised by MNN-V, SSS is a collaborator of the group.

## Supplementary Material

Additional file 1: Figure S1 Treating of HTB2-m Cherry S. cerevisiae strain with various amounts of detergent. **Figure S2.** Cells continue the cell cycle progression in the presence of HU, but the duration of the cell cycle is prolonged. Click here for file

Additional file 2: Movie S2 Live cell imaging of Mrc1-GFP in 100 mM HU. Time-lapse live cell imaging of *MRC1-GFP* yeast cells in the presence of 100 mM HU. Click here for file

Additional file 3: Movie S4 Live cell imaging of Rad53-GFP in 100 mM HU. Time-lapse live cell imaging of *Rad53-GFP* yeast cells in the presence of 100 mM HU. Click here for file

Additional file 4: Movie S1 Live cell imaging of Mrc1-GFP. Time-lapse live cell imaging of *MRC1-GFP* yeast cell throughout the cell cycle. Click here for file

Additional file 5: Movie S3 Live cell imaging of Rad53-GFP. Time-lapse live cell imaging of *Rad53-GFP* yeast cell throughout the cell cycle. Click here for file
