# Supplementary material for: The subunits of the S-phase checkpoint complex Mrc1/Tof1/Csm3: dynamics and interdependence
Source: Cell Div. 2014 Oct 31;9:4. doi: 10.1186/1747-1028-9-4 (PMC4221646; doi:10.1186/1747-1028-9-4)
Supplement: Additional file 1: Figure S1 — Treating of HTB2-m Cherry S. cerevisiae strain with various amounts of detergent. Figure S2. Cells continue the cell cycle progression in the presence of HU, but the duration of the cell cycle is prolonged. [file 1747-1028-9-4-S1.docx]

**
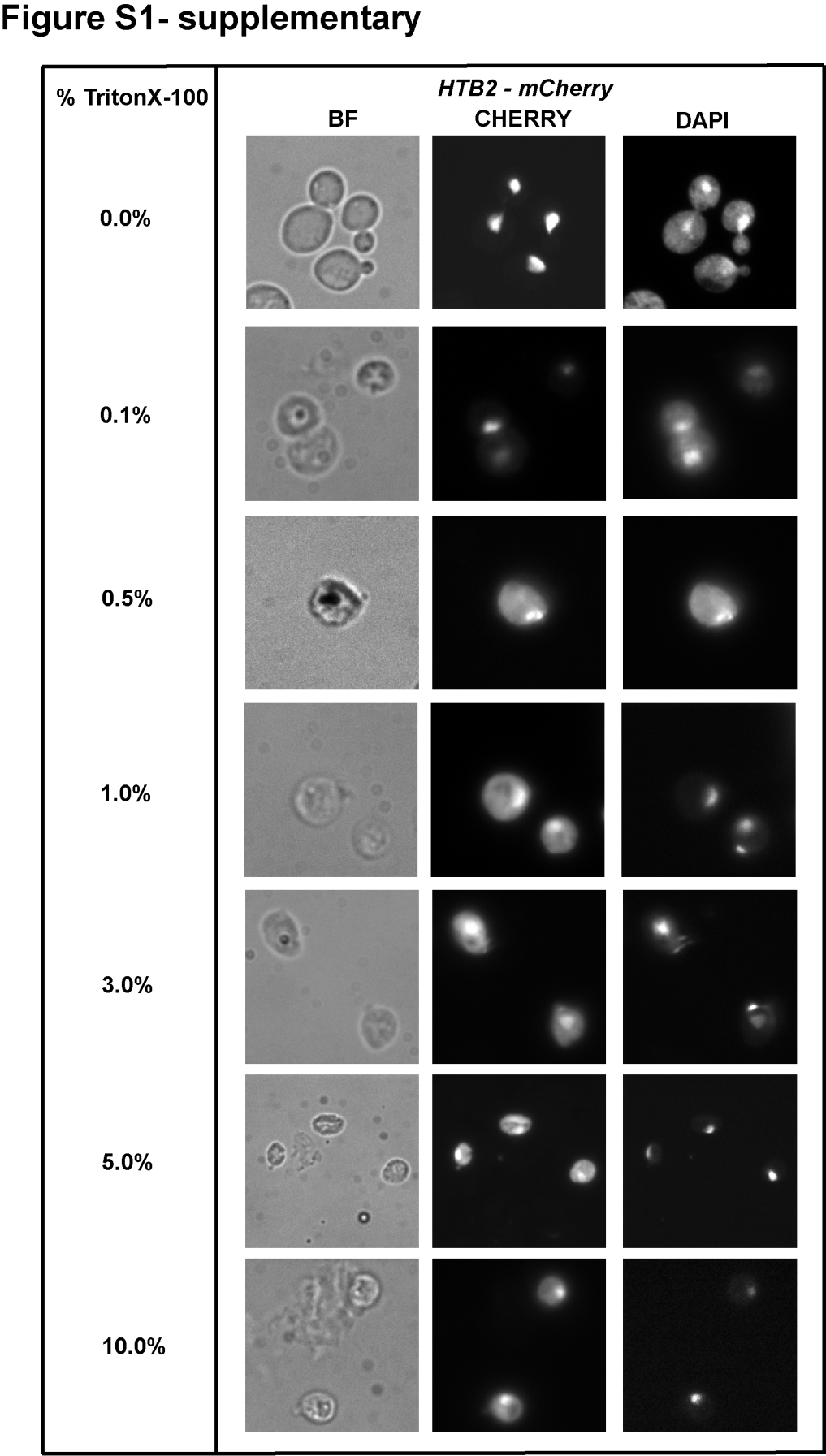
Fig. S1. Treating of *HTB2-mCherry* S. cerevisiae strain with various amounts of detergent.** After partial spheroplasting by LongLife^TM^ Zymolyase®, the control *HTB2-mCherry* strain is subjected to soluble proteins washing via TritonX-100 detergent treatment. To determine the amount of detergent that eliminates soluble proteins, but is not affecting chromatin bound molecules, various amounts of TritonX-100 are tested (0.1% - 10.0% w/v). Cells are paraformaldehyde fixed and subjected to fluorescent microscopy analysis to detect the position of mCherry-tagged proteins. 2.5 µg/ml DAPI staining is used for all probes to visualize the position of DNA. The obtained mCherry and DAPI signals are analyzed for co-localizations. Higher percentages of detergent lead to reduction of the amount of intact cells left in the probe. Interestingly, even when 10.0% of TritonX-100 is applied, cells that still kept their integrity, exhibited match of mCherry and DAPI signals. As the interaction between histones and DNA are known to be among the strongest, the lack of elimination of HTB2-mCherry signal was not surprising.


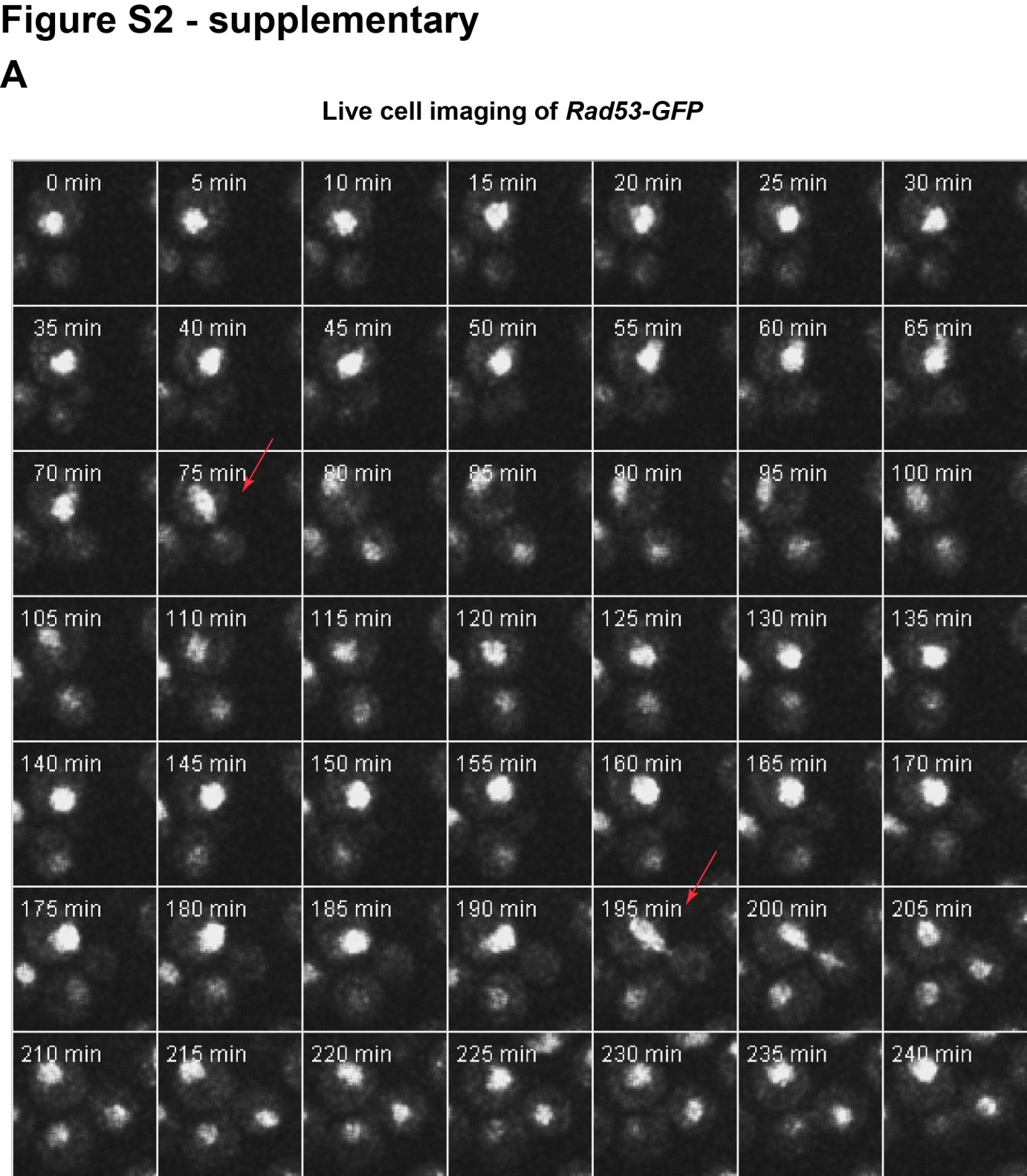


­


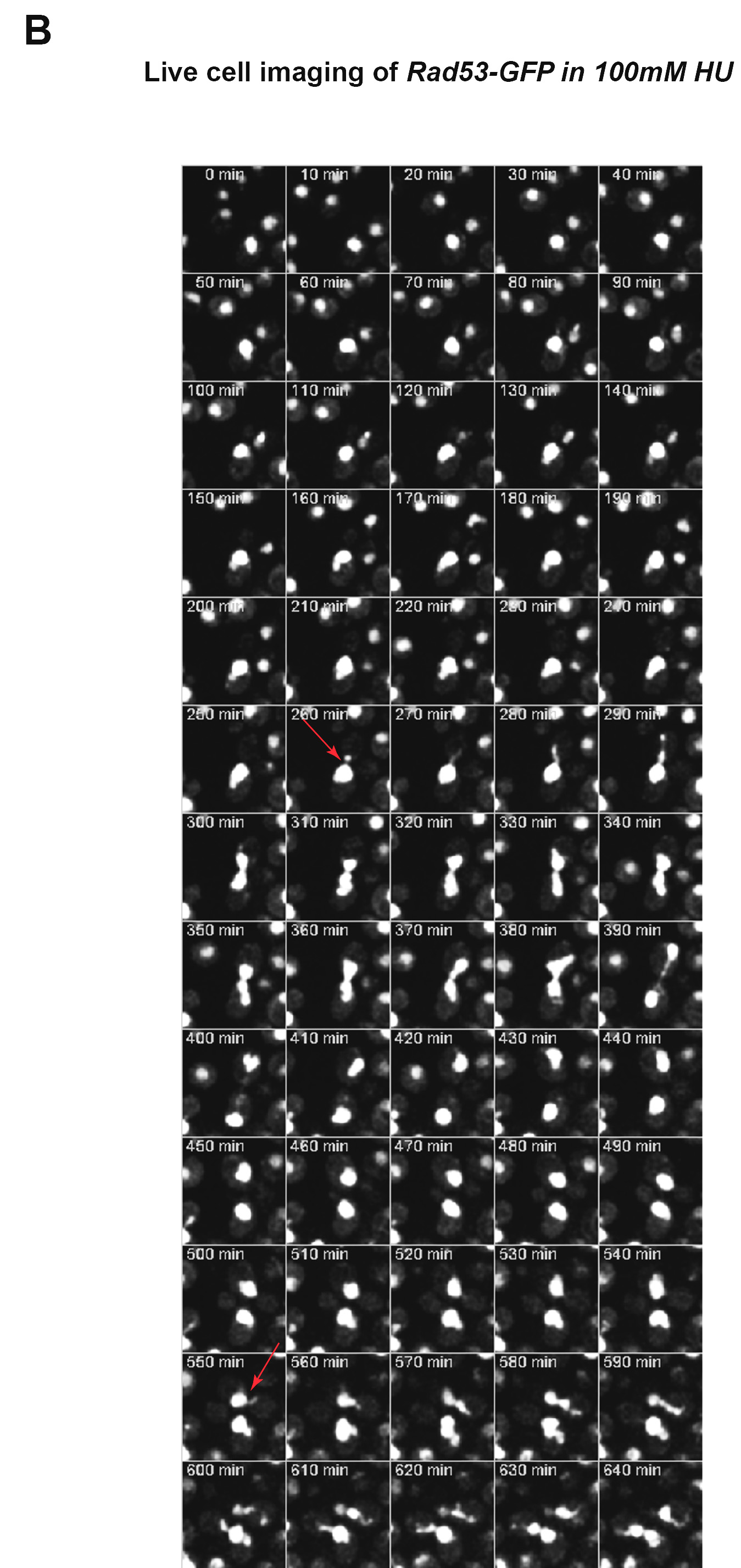

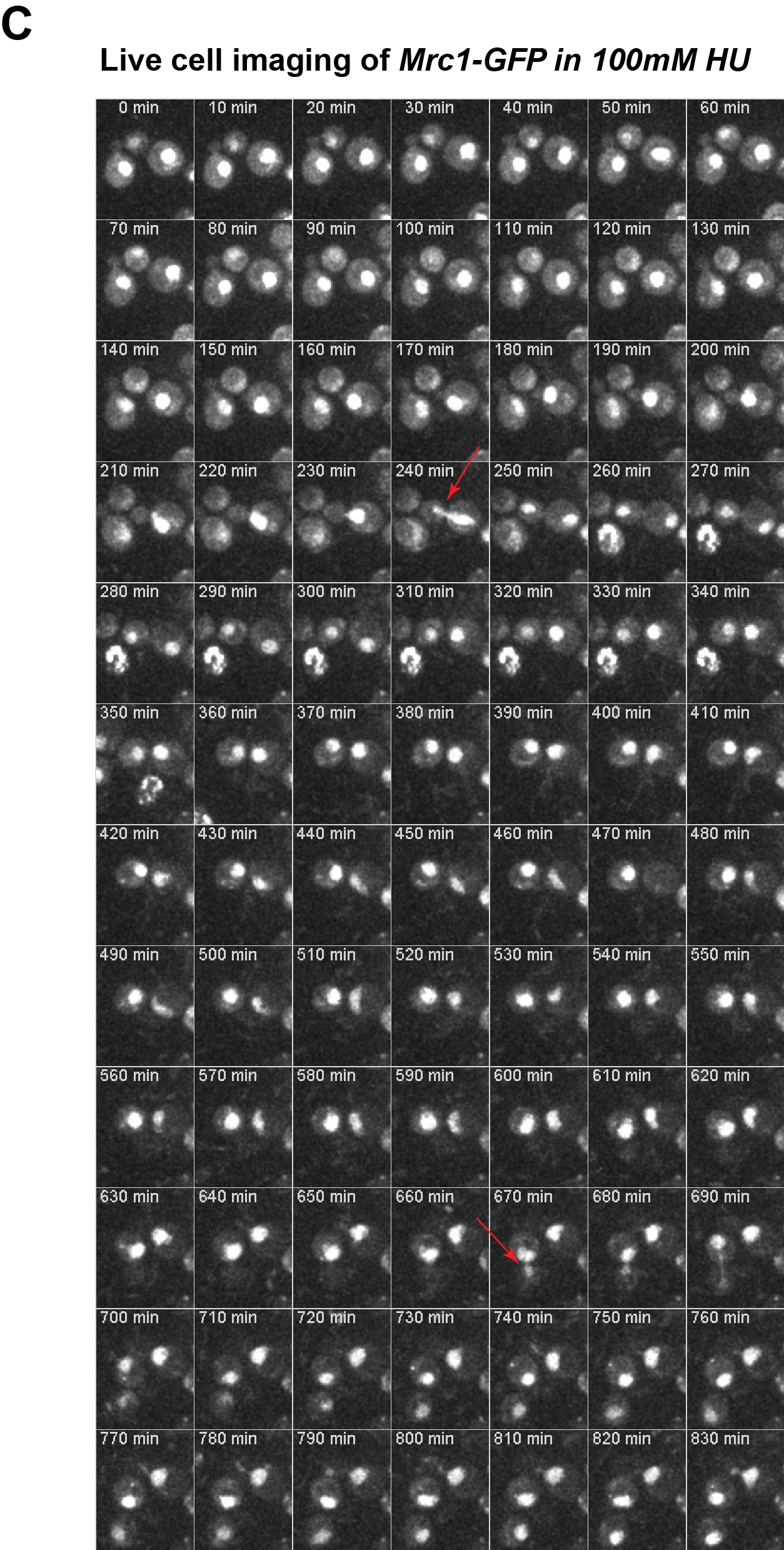


**Fig. S2. Cells continue the cell cycle progression in the presence of HU, but the duration of the cell cycle is prolonged. (A.)** Time-lapse live cell imaging of *Rad53-GFP* yeast cell throughout the cell cycle.

**(B.)** Time-lapse live cell imaging of *Rad53-GFP* yeast cell in 100mM HU.

**(C.)** Time-lapse live cell imaging of *Mrc1-GFP* yeast cell in 100mM HU.

The arrows indicate the characteristic anaphase nuclear morphology of the cell. The frames situated between two arrows comprise of one entire cell cycle.

All time-lapse live cell imaging experiments are also presented as movies:

**Movie 1:** Live cell imaging of Mrc1-GFP

**Movie 2:** Live cell imaging of Mrc1-GFP in 100 mM HU

**Movie 3:** Live cell imaging of Rad53-GFP

**Movie 4:** Live cell imaging of Rad53-GFP in 100 mM HU
